# Acoustic field induced nonlinear magneto-optical rotation in a diamond mechanical resonator

**DOI:** 10.1038/s41598-020-65049-2

**Published:** 2020-05-18

**Authors:** Mohsen Ghaderi Goran Abad, Fatemeh Ashrafizadeh Khalifani, Mohammad Mahmoudi

**Affiliations:** Department of Physics, University of Zanjan, University Blvd., 45371-38791 Zanjan, Iran

**Keywords:** Magneto-optics, Nonlinear optics

## Abstract

We study the nonlinear magneto-optical rotation (MOR) of a linearly polarized microwave probe field passing through many nitrogen-vacancy (NV) centers embedded in a high-Q single-crystal diamond mechanical resonator. On the basis of the strain-mediated coupling mechanism, we establish a three-level closed-loop system in the ground states of the NV center in the presence of a static magnetic field. It is shown that by applying an acoustic field, the birefringence is induced in the system through the cross-Kerr effect, so that the probe field is transmitted with a high intensity and rotated polarization plane by 90 degrees. In addition, we demonstrate that the acoustic field has a major role in enhancing the MOR angle to 90 degrees. Moreover, it is shown that the MOR angle of the polarization plane after passing through the presented system is sensitive to the relative phase of the applied fields. The physical mechanism of the MOR enhancement is explained using the analytical expressions which are in good agreement with the numerical results. The presented scheme can be used as a polarization converter for efficient switching TE/TM modes in optical communication, the depolarization backscattering lidar, polarization spectroscopy and precision measurements.

## Introduction

Light-matter interaction in the presence of a magnetic field gives rise to the magneto-optical effects. Polarization rotation of light in the presence of a magnetic field is one of the most well-known among the magneto-optical effects, which has been attracting many interests for many decades. Polarization -specifying the geometrical orientation of the electric field oscillations- is one of the essential features of light, which its recognition and manipulation play an important role in theoretical and experimental studies in the light-matter interaction^1,2^. In fact, a linearly polarized light experiences polarization rotation during passing through an asymmetric medium induced by a static magnetic field known as linear magneto-optical rotation (MOR). Magnetic field can rotate the polarization plane which is known as Faraday effect^3^ or Voigt effect^4^, depending on the propagation of the linearly polarized light through a medium placed in a longitudinal or transverse static magnetic field, respectively. In nonlinear MOR, the asymmetry is induced by applying laser fields as well as a static magnetic field. The asymmetry makes a difference between refractive indices of the left- and right- circular components of the linearly polarized field, leading to the rotation of the polarization plane. It has been shown that the combination of the magnetic field and control laser fields can enhance the polarization rotation. Numerous studies have been theoretically and experimentally done on the MOR of the polarization plane in various wavelength regions in atomic gasses^5–8^, GaAs quantum well waveguide^9^, metamaterials^10,11^ and graphene^12,13^. Magneto-optical rotation has found many applications^14^ in magnetometery^15–17^, optical limiting^18,19^, optical filters^20–23^ and atomic clocks^24^.

Here, we are going to use the nitrogen-vacancy (NV) centers to generate the MOR. Nitrogen-vacancy center consists of a nitrogen atom instead of a carbon atom inside a host diamond lattice, which gives diamond a yellow color. In other word, the NV center is a point defect center in a solid state diamond with long coherence time and optical addressability at room temperature^25,26^. Because of its excellent coherence properties and its ability to be coherently coupled to various external fields, it has provided a promising candidate for quantum information processing^27,28^ and quantum physics experiments. Moreover, electronic energy-level structure of the NV center includes a spin-triplet ground state which can be coherently excited using microwave fields, so that the NV center in diamond makes it possible to study the quantum dynamic of spin states^29^.

Although the MOR has been investigated in three-level quantum systems^30–32^, to the best of our knowledge, the MOR has not been observed in optically generated closed-loop three-level quantum system. It is well known that the electric dipole transition rules of the quantum levels play an important role in transitions of an atomic system. This makes a limitation in establishing the closed-loop schemes in degenerate levels of an atomic system. The limitation has been removed by using the strain-mediated coupling mechanism along with microwave field in the NV centers in a diamond mechanical resonator (DMR). Diamond mechanical resonator includes a diamond cantilever with many embedded NV centers, which can interact with the resonant phonon modes of a mechanical resonator through the crystal strain. It is a device with micron-scale dimensions, which can be achieved with excellent nanofabrication techniques in experiment^33^. The strain field is produced by the lattice vibration via a piezoelement used on the surface of the diamond layer and transferred through the DMR to couple the lattice strain field and the NV center spins. Many investigations have demonstrated the coherently coupling of the ground triplet state of the NV centers with the strain field in the DMR^34–37^. Transparency for the acoustic field using the ground triplet state of the NV center driven by the strain and microwave fields has been reported in either V- or Δ-type configuration^38^. Evangelou showed the phase dependent of the transparency of the acoustic field by regarding the Δ-type configuration of the NV center ground state as a closed-loop system^39^. Earlier, Fuchs *et al*.^36^ had demonstrated the sensitivity of Δ-system of the NV center ground state to the relative phase of the applied fields and strain field made in the DMR. Magneto-optical rotation in a tripod four-level NV centers has been previously reported in which the ground triplet state ^3^A is coupled to the excited state ^3^E via visible optical fields^40^. Here, we use the DMR as a source of the strain field to excite the electric dipole forbidden transition of the system to generate the complete MOR.

Now, we take advantage of coupling the ground triplet state of NV center with the strain field and study the MOR of the polarization plane of a linearly polarized microwave probe field in a three-level closed-loop system of NV center’s ground state in the DMR. This work is aimed to bring the optical phenomena into the acoustic field’s domain. It is presented that by applying an acoustic strain field in the presence of a static magnetic field, difference between the refractive indices of the circular components of the probe field increases and the birefringence is induced in the system. We show that the linearly polarized microwave probe field is transmitted through the system with high intensity while its polarization plane is rotated by 90 degrees. It is demonstrated that applying the acoustic field enhances the MOR angle of the polarization plane of the probe field due to the cross-Kerr effect. In addition, we show that the MOR angle of the polarization plane of the probe field is sensitive to the relative phase of the applied fields and the perfect rotation of the polarization plane happens for the special values of the relative phase. Our analytical results show that the nonlinear cross-Kerr effect is the responsible for the MOR enhancement. The obtain results can be used in the TE/TM polarization modes converters in optical communication, the depolarization backscattering lidar, polarization spectroscopy and precision measurements.

## Theoretical framework

**Figure 1:**
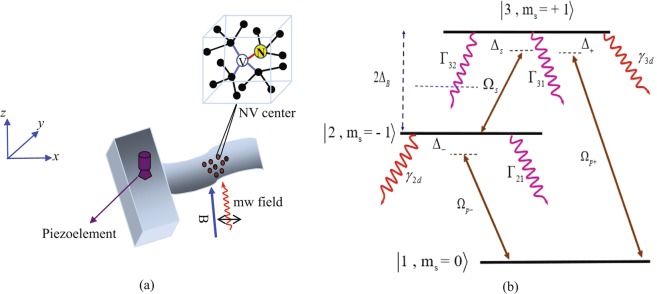
(**a**) The system under consideration is a DMR with many embedded NV centers. (**b**) Diagram of the three-level closed-loop of the NV center ground state. The transitions $\mathrm{|1}\rangle$ to $\mathrm{|2}\rangle$ and $\mathrm{|3}\rangle$ are driven by the left- and right- circularly polarized microwave probe field with Rabi frequency ${\Omega }_{p-}$ and ${\Omega }_{p+}$, respectively. In addition, the electric dipole forbidden transition $\mathrm{|2}\rangle$ to $\mathrm{|3}\rangle$ is coupled by the strain field with Rabi frequency ${\Omega }_{s}$.

The system under study is composed of a high-Q single-crystal DMR with many embedded NV centers which is shown in Fig. 1(a). The NV centers are sensitive to the deformation of the surrounding lattice. When the DMR is vibrated by a piezoelectric film, the strain field is formed and transferred to wherever the NV centers are located. It is noteworthy that the strain in the DMR can be controlled by an external voltage applied to the piezoelectric film. The NV centers considered in this work are negatively charged with two unpaired electrons located at the vacancy. Thus, their ground state has a spin-triplet form. The schematic of the three-level closed-loop system of the NV center’s ground state is shown in Fig. 1(b). The spin state ${|}^{3}A,{m}_{s}=0\rangle$ (labeled by $\mathrm{|1}\rangle$) experiences a zero-field splitting by 2.87 GHz from the degenerate spin states ${|}^{3}A,{m}_{s}=\pm \,1\rangle$(labeled by $|\pm 1\rangle$) due to the spin-spin interaction. A linearly polarized microwave weak probe field $\overrightarrow{E}=\hat{x}{E}_{p}exp[-i({\omega }_{p}t-{k}_{p}z)]+c\mathrm{}.c$ is applied to the system parallel to the static magnetic field known as Faraday geometry. Since a linearly polarized field is a combination of right- and left- circularly polarized field, the right- and left- circular components of the probe field excite the transition $\mathrm{|1}\rangle \leftrightarrow \mathrm{|3}\rangle$ and $\mathrm{|2}\rangle \leftrightarrow \mathrm{|3}\rangle$ with Rabi frequencies ${\Omega }_{p+}=({\overrightarrow{\mu }}_{31}\mathrm{}.{\hat{\varepsilon }}_{+}){E}_{+}/\hslash$ and ${\Omega }_{p-}=({\overrightarrow{\mu }}_{21}\mathrm{}.{\hat{\varepsilon }}_{-}){E}_{-}/\hslash$, respectively. The transition $\mathrm{|2}\rangle \leftrightarrow \mathrm{|3}\rangle$ is electric dipole forbidden ($\Delta m=2$). However, this transition can be coherently coupled by the strain field made by the lattice vibration with Rabi frequency ${\Omega }_{s}=({\overrightarrow{\mu }}_{32}\mathrm{}.{\hat{\varepsilon }}_{s}){E}_{s}/\hslash$. Thus, a three-level closed-loop system is formed by the strain field in the degenerate ground state of the NV center. Here, ${E}_{p}$ and ${E}_{s}$ are the amplitudes of the probe field and strain field, respectively. Also, ${\hat{\varepsilon }}_{i}(i=\pm ,s)$ are the unit polarization vector of the circular components of the linear probe field and unit vector of the strain field. In addition, we have ${E}_{+}={E}_{-}={E}_{p}/\sqrt{2}$ and $|{\overrightarrow{\mu }}_{31}|=|{\overrightarrow{\mu }}_{32}|$. Since displacements of the electrons density of lattice induced by strain wave lead to generate a local electric field, we model the strain field as an effective electric field. By applying a static magnetic field, the degeneracy of the $|\pm 1\rangle$ can be removed by 2 $\hslash \Delta B=2{m}_{s}{g}_{s}{\mu }_{B}B$ where ${\mu }_{B}$ and ${g}_{s}$ are Bohr magneton and Land $e{\prime}$ factor, respectively. ${m}_{s}$ stands for magnetic quantum number of the states.

The Hamiltonian describing the interaction between two circularly polarized fields and strain field in the rotating wave and dipole approximations is given by^41^

1 $${V}_{I}=-\,\hslash ({\Omega }_{p-}{e}^{-i({\Delta }_{-}+{\Delta }_{B})t}\mathrm{|2}\rangle \langle \mathrm{1|}+{\Omega }_{p+}{e}^{-i({\Delta }_{+}-{\Delta }_{B})t}\mathrm{|3}\rangle \langle \mathrm{1|}+{\Omega }_{s}{e}^{-i({\Delta }_{c}-2{\Delta }_{B})t}\mathrm{|3}\rangle \langle \mathrm{2|)}+h\mathrm{}.c\mathrm{.,}$$

where ${\Delta }_{-}={\omega }_{p-}-{\omega }_{21}$, ${\Delta }_{+}={\omega }_{p+}-{\omega }_{31}$ and ${\Delta }_{s}={\omega }_{s}-{\omega }_{32}$ are the detunings of the applied fields from the corresponding transitions.

Using the von Neumann equation^41^, the density matrix equations of motion for the three-level closed-loop system in the electric-dipole and rotating-wave approximations can be written as

2 $$\begin{array}{c}{\dot{\rho }}_{22}={\Gamma }_{32}{\rho }_{33}-{\gamma }_{2}{\rho }_{22}+i{\Omega }_{p-}^{\ast }{\rho }_{12}+i{\Omega }_{s}{e}^{-i\Phi }{\rho }_{32}-i{\Omega }_{p-}{\rho }_{21}-i{\Omega }_{s}^{\ast }{e}^{i\Phi }{\rho }_{23},\\ {\dot{\rho }}_{33}=-\,{\gamma }_{3}{\rho }_{33}+i{\Omega }_{s}^{\ast }{e}^{i\Phi }{\rho }_{23}+i{\Omega }_{p+}^{\ast }{\rho }_{13}-i{\Omega }_{s}{e}^{-i\Phi }{\rho }_{32}-i{\Omega }_{p+}{\rho }_{31},\\ {\dot{\rho }}_{12}=-\,(\frac{{\gamma }_{2}}{2}+i({\Delta }_{-}+{\Delta }_{B})){\rho }_{12}+i{\Omega }_{p-}({\rho }_{22}-{\rho }_{11})+i{\Omega }_{p+}{e}^{i\varDelta t}{\rho }_{32}-i{\Omega }_{s}{e}^{i(\Delta t+\Phi )}{\rho }_{13},\\ {\dot{\rho }}_{13}=-\,(\frac{{\gamma }_{3}}{2}+i({\Delta }_{+}-{\Delta }_{B})){\rho }_{13}+i{\Omega }_{p+}({\rho }_{33}-{\rho }_{11})+i{\Omega }_{p-}{e}^{-i\Delta t}{\rho }_{32}+{\Omega }_{s}{e}^{i(-\Delta t+\Phi )}{\rho }_{12},\\ {\dot{\rho }}_{23}=-\,((\frac{{\gamma }_{2}}{2}+\frac{{\gamma }_{3}}{2})+i({\Delta }_{s}-2{\Delta }_{B})){\rho }_{23}+i{\Omega }_{s}{e}^{-i\Phi }({\rho }_{33}-{\rho }_{22})+i{\Omega }_{p-}^{\ast }{e}^{i\Delta t}{\rho }_{13}-i{\Omega }_{p+}^{\ast }{e}^{i\Delta t}{\rho }_{12},\\ {\dot{\rho }}_{11}=-\,({\dot{\rho }}_{22}+{\dot{\rho }}_{33}),\end{array}$$

where ${\Gamma }_{3i}$(i = 1,2) and ${\Gamma }_{21}$ are the spontaneous emission from the state $\mathrm{|3}\rangle$ and $\mathrm{|2}\rangle$ to $|i\rangle$ and $\mathrm{|1}\rangle$, respectively. In addition, ${\gamma }_{2}={\Gamma }_{21}+{\gamma }_{2d}$, ${\gamma }_{3}={\gamma }_{3d}+{\Gamma }_{3}$, ${\Gamma }_{3}={\Gamma }_{31}+{\Gamma }_{32}$, where ${\gamma }_{3d}$ (${\gamma }_{2d}$) is the dephasing rate of the state $\mathrm{|3}\rangle$ ($\mathrm{|2}\rangle$). $\Delta ={\Delta }_{+}-{\Delta }_{-}-{\Delta }_{s}$ is the multi-photon resonance detuning. The response of the quantum system to the right- and left- circularly polarized probe field is described by the susceptibility, which is given by^5^

3 $${\chi }_{\pm }=(\frac{\alpha }{4\pi {k}_{p}}){S}_{\pm }\mathrm{}.$$

Here, $\alpha l=4\pi {k}_{p}l{\mu }^{2}N/\hslash \gamma$ is the field absorption at resonance in which $l$, ${k}_{p}$ and $N$ are the length of the atomic medium, probe field wave number and density of atoms, respectively. S_±_ are the normalized susceptibilities which are given by

4 $${S}_{+}=\frac{{\rho }_{31}{\gamma }_{31}}{{\Omega }_{p+}},\,{S}_{-}=\frac{{\rho }_{21}{\gamma }_{21}}{{\Omega }_{p-}},$$

where ${\rho }_{i1}$ (i = 3,2) is the probe field transition coherence, which can be obtained from Eq. (2) The imaginary and real parts of S_±_ represent the absorption and dispersion of the circularly polarized probe field, respectively. The polarization direction of the input linearly polarized probe field is assumed in $\hat{x}$ direction, which may be affected and rotated after passing through a medium. If a component of the polarization direction of the output probe field is observed in $\hat{y}$ direction, the polarization plane of the probe field has been rotated. In experiment, a y-polarized analyzer is used to transmit the light with polarization in $\hat{y}$ direction. Thus, the intensity of transmission in $\hat{y}$ direction is used to calculate the polarization rotation of the output probe field. The intensity of transmission of the light with $\hat{y}$ and $\hat{x}$ polarization directions are given by^5^

5 $${T}_{y}=\frac{|({E}_{{p}_{(out)}}{)}_{y}{|}^{2}}{|{E}_{{p}_{(in)}}{|}^{2}}=\frac{1}{4}|\exp [i\alpha l{S}_{+}\mathrm{/2]}-\exp [i\alpha l{S}_{-}{\mathrm{/2]|}}^{2}$$

6 $${T}_{x}=\frac{|({E}_{{p}_{(out)}}{)}_{x}{|}^{2}}{|{E}_{{p}_{(in)}}{|}^{2}}=\frac{1}{4}|\exp [i\alpha l{S}_{+}\mathrm{/2]}+\exp [i\alpha l{S}_{-}{\mathrm{/2]|}}^{2}\mathrm{}.$$

The MOR angle of the probe field polarization is defined as

7 $$\phi =ta{n}^{-1}[\sqrt{{T}_{y}/{T}_{x}}\mathrm{]}.$$

The polarization rotation of a laser field after passing through a medium can occur due to the birefringence or dichroism induced in the system. When the dispersions (absorptions) of the right- and left- circular components of the linearly polarized probe field are different, while their absorptions (dispersions) are equal, the birefringence (dichroism) is dominant in the system. By separating the real and imaginary parts of ${S}_{+}$ and ${S}_{-}$ in Eqs. (5) and (6) and applying the mere birefringence conditions $Re[{S}_{+}]\ne Re[{S}_{-}]$ and $Im[{S}_{+}]=Im[{S}_{-}]=\beta$, these equations take the form as

8 $${T}_{y}=\frac{{e}^{-\alpha l\beta }}{4}|\exp [i\alpha lRe[{S}_{+}\mathrm{]/2]}-\exp [i\alpha lRe[{S}_{-}{\mathrm{]/2]|}}^{2},$$

9 $${T}_{x}=\frac{{e}^{-\alpha l\beta }}{4}|\exp [i\alpha lRe[{S}_{+}\mathrm{]/2]}+\exp [i\alpha lRe[{S}_{-}{\mathrm{]/2]|}}^{2}\mathrm{}.$$

When $\beta$ is positive but negligible, what we were looking for in our results, the rotation of the polarization direction of the probe field is merely due to the birefringence induced in the system without any attenuation of the intensity of the probe field after passing through the medium. The negative values of $\beta$ show the amplification of the intensity of the probe field.

## Results and Discussion

**Figure 2:**
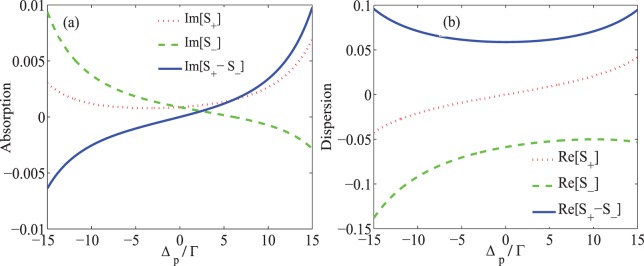
The imaginary (**a**) and real (**b**) parts of ${S}_{+}$ (dotted) and ${S}_{-}$ (dashed) and their difference (solid) versus detuning of the probe field ${\Delta }_{p}$. The used parameters are ${\Omega }_{p}=0.01\Gamma$, ${\Omega }_{s}=17\Gamma$, ${\Delta }_{B}=17\Gamma$, ${\Gamma }_{31}={\Gamma }_{32}={\gamma }_{3d}=\Gamma$, ${\Gamma }_{21}={\gamma }_{2d}=0.01\Gamma$, $\alpha l=107\Gamma$, ${\Delta }_{c}=0$ and $\Phi =0$.

In this section, we are going to investigate the MOR in the three-level closed-loop quantum system established in the NV center’s ground state affected by the strain field in the DMR. Throughout the results, it is assumed that ${\Delta }_{-}={\Delta }_{+}={\Delta }_{p}$, ${\Omega }_{p-}={\Omega }_{p+}={\Omega }_{p}$ and $\Delta =0$. Also, the parameters are scaled by ${\Gamma }_{31}={\Gamma }_{32}={\gamma }_{3d}=\Gamma$, which is equal to $2.2MHz$^36^. Figure 2 shows the imaginary (a) and real (b) parts of ${S}_{+}$ (dotted) and ${S}_{-}$ (dashed) describing the behavior of the absorption and dispersion of the right- and left- circular components of the probe field and their difference (solid) versus the detuning of the probe field. The taken parameters are ${\Omega }_{p}=0.01\Gamma$, ${\Omega }_{s}=17\Gamma$, ${\Delta }_{B}=17\Gamma$, ${\Gamma }_{21}={\gamma }_{2d}=0.01\Gamma$, $\alpha l=107\Gamma$, ${\Delta }_{c}=0$ and $\Phi =0$. Note that ${\Delta }_{B}=17\Gamma$ is corresponding to $2Gauss$ static magnetic field. An investigation on Fig. 2(a) shows that the absorption of the circular components of the probe field is equal and negligible around the probe field resonance. Figure 2(b) shows that the corresponding dispersions difference is noticeable at ${\Delta }_{p}=0$. It represents that the difference between the normalized susceptibilities, ${S}_{+}$ and ${S}_{-}$, happens only due to the difference between the refractive indices (dispersions) of the right- and left- circular components of the probe field. Thus, the birefringence generated by the difference of the real parts of the normalized susceptibilities is a merely dominant phenomenon in the system.

**Figure 3:**
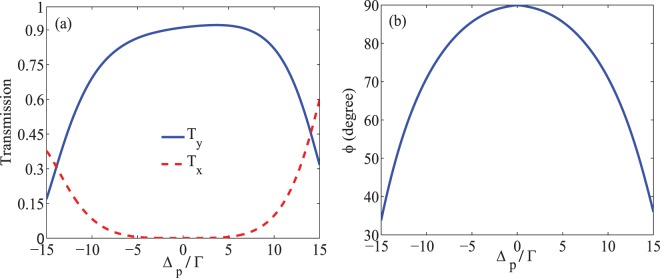
Intensity of the transmission (**a**) in direction $\hat{x}$ (dashed) and $\hat{y}$ (solid) and the MOR angle (**b**) versus detuning of the probe field. The taken parameters are the same used in Fig. 2.

In Fig. 3, the $\hat{x}$-(dashed) and $\hat{y}$-(solid) components of the transmitted field (a) as well as the MOR angle of the polarization direction of the probe field (b) are presented versus the detuning of the probe field. The parameters are those used in Fig. 2. It is seen in Fig. 3(a) that the intensity of the transmitted field with rotated polarization, ${T}_{y}$, increases by 0.91 at ${\Delta }_{p}=0$, while ${T}_{x}$, the intensity of the transmitted field with primary polarization direction, attenuates extremely. Since the primary polarization of the probe field is considered in $\hat{x}$ direction, measuring a large transmission in $\hat{y}$ direction indicates a complete rotation of the polarization direction of the probe field passing through the medium. Moreover, it is seen in Fig. 3(b) that the MOR angle value reaches 90 degrees at ${\Delta }_{p}=0$, which means that the polarization plane of the probe field has been completely rotated after passing through the quantum system. It is worth noting that the maximum MOR happens merely due to the induced birefringence as shown in Fig. 2.

**Figure 4:**
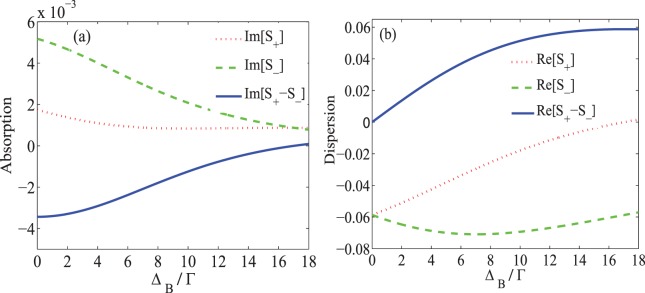
The imaginary (**a**) and real (**b**) parts of ${S}_{+}$ (dotted) and ${S}_{-}$ (dashed) and their difference (solid) versus ${\Delta }_{B}$. The taken parameters are ${\Omega }_{p}=0.01\Gamma$, ${\Omega }_{s}=17\Gamma$, ${\Gamma }_{31}={\Gamma }_{32}={\gamma }_{3d}=\Gamma$, ${\Gamma }_{21}={\gamma }_{2d}=0.01\Gamma$, $\alpha l=107\Gamma$, ${\Delta }_{c}=0$, $\Phi =0$ and ${\Delta }_{p}=0$.

**Figure 5:**
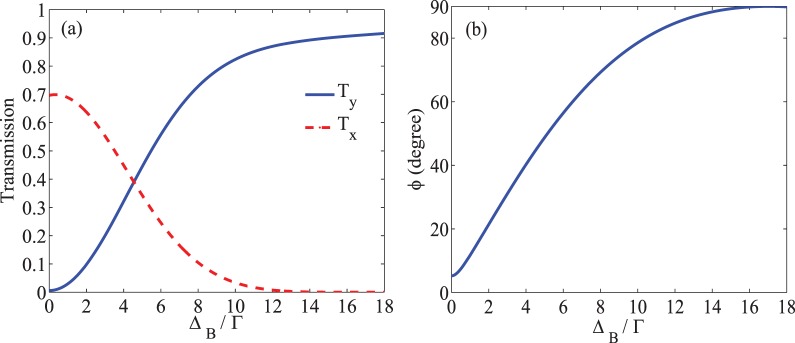
Intensity of the transmission (**a**) in $\hat{x}$ (dashed) and $\hat{y}$ (solid) directions and the MOR angle (**b**) versus ${\Delta }_{B}$. The taken parameters are the same used in Fig. 4.

Another parameter for controlling the MOR angle is the static magnetic field which causes the Zeeman splitting of the energy levels. The static magnetic field can be applied to the NV centers by placing the DMR in center of the Helmholtz coils and controlled by the input DC current. The effect of the static magnetic field on the MOR is displayed in Figs. 4 and 5. Figure 4 shows the absorption (a) and dispersion (b) of the right- (dotted), left- (dashed) circular polarization of probe field and their difference (solid) versus Zeeman splitting ${\Delta }_{B}$ in the probe field resonance condition. The used parameters are ${\Omega }_{p}=0.01\Gamma$, ${\Omega }_{s}=17\Gamma$, ${\Gamma }_{31}={\Gamma }_{32}={\gamma }_{3d}=\Gamma$, ${\Gamma }_{21}={\gamma }_{2d}=0.01\Gamma$, $\alpha l=107\Gamma$, ${\Delta }_{c}=0$ and $\Phi =0$. It is seen in Fig. 4 that the dichroism is dominant in the system in the absence of the static magnetic field. By increasing the static magnetic field, one can see that the absorption of the circular components of the probe field and their difference dramatically decrease, while the dispersion of the right- and left- circular polarization of the probe field and their difference grow to the maximum value. It is expected that the contribution of dichroism and birefringence in the MOR is completely dependent on the magnitude of the static magnetic field.

In Fig. 5, the intensity of transmission of the output probe field (a) in $\hat{x}$ (dashed) and $\hat{y}$ (solid) directions and the MOR angle of the polarization direction of the probe field (b) are plotted versus ${\Delta }_{B}$. It is shown in Fig. 5(a) that in the absence of the static magnetic field, the y-component of the transmitted probe field is negligible. Also, the transmission of the probe field with primary polarization, accompanied by higher absorption, is smaller than 0.7. It is illustrated that by increasing the static magnetic field, ${T}_{y}$ increases and reaches a maximum value at ${\Delta }_{B}=17\Gamma$, while ${T}_{x}$ attenuates. According to Fig. 5(b), by increasing the static magnetic field, the MOR angle of the polarization direction of the probe field increases and experiences a rotation by 90 degrees at ${\varDelta }_{B}=17\Gamma$. It can be said that increasing the static magnetic field enhances the MOR angle by increasing the difference between the refractive indices of the circular components of the probe field.

**Figure 6:**
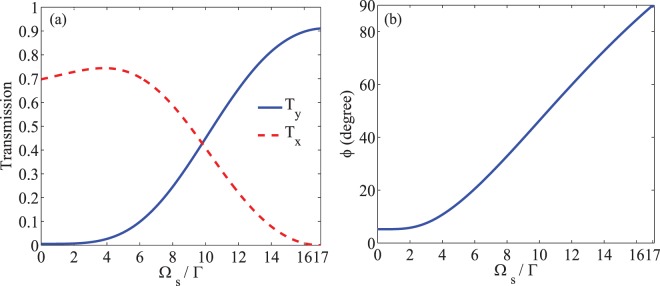
Intensity of the transmission (**a**) in $\hat{x}$ (dashed) and $\hat{y}$ (solid) directions and MOR angle (**b**) versus intensity of the acoustic field. The used parameters are ${\Omega }_{p}=0.01\Gamma$, ${\Delta }_{B}=17\Gamma$, ${\Gamma }_{31}={\Gamma }_{32}={\gamma }_{3d}=\Gamma$, ${\Gamma }_{21}={\gamma }_{2d}=0.01\Gamma$, $\alpha l=107\Gamma$, ${\Delta }_{c}=0$, $\Phi =0$ and ${\Delta }_{p}=0$.

The next scenario is controlling the MOR via the acoustic field. Noting that the strain field is produced by means of a piezoelement attached to the DMR, the intensity of the acoustic field can be well controlled by the external voltage applied to the piezoelement. Figure 6 displays the effect of the acoustic field on the MOR. In Fig. 6, the behavior of the ${T}_{x}$ (dashed), ${T}_{y}$ (solid) (a) and MOR angle (b) of the probe field are plotted versus the intensity of the acoustic field ${\varOmega }_{s}$ at ${\Delta }_{p}=0$. The used parameters are ${\Omega }_{p}=0.01\Gamma$, ${\Delta }_{B}=17\Gamma$, ${\Gamma }_{31}={\Gamma }_{32}={\gamma }_{3d}=\Gamma$, ${\Gamma }_{21}={\gamma }_{2d}=0.01\Gamma$, $\alpha l=107\Gamma$, ${\Delta }_{c}=0$ and $\Phi =0$. A bird’s eye view of Fig. 6(a) shows that the $\hat{y}$-component of the output probe field is zero in the absence of the acoustic field, but ${T}_{y}$ enhances by increasing the intensity of the acoustic field, while ${T}_{x}$ decreases. It is noteworthy that the birefringence due to the dispersion difference of two probe field components has a major role in establishing the local maximum in ${T}_{x}$. Figure 6(b) demonstrates the effective role of the acoustic strain field in enhancing the MOR of the polarization plane of the probe field. It is seen that by switching off the acoustic field, the MOR angle due to the linear response of the medium becomes negligible. By increasing the intensity of the acoustic field, the nonlinear optical effects enhance the MOR angle and brings it to the maximum value.

**Figure 7:**
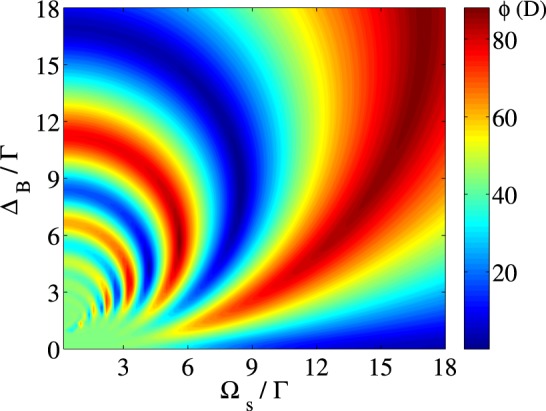
MOR angle of the polarization direction of the linearly polarized probe field versus ${\Omega }_{s}$ and ${\Delta }_{B}$. The other taken parameters are ${\Omega }_{p}=0.01\Gamma$, ${\Gamma }_{31}={\Gamma }_{32}={\gamma }_{3d}=\Gamma$, ${\Gamma }_{21}={\gamma }_{2d}=0.01\Gamma$, $\alpha l=107\Gamma$, ${\Delta }_{c}=0$, ${\Delta }_{p}=0$ and $\Phi =0$.

The simultaneous effect of the static magnetic field and the acoustic field on the MOR angle of the polarization plane of the linearly polarized field at ${\Delta }_{p}=0$ is shown in Fig. 7. The other used parameters are ${\Omega }_{p}=0.01\Gamma$, ${\Gamma }_{31}={\Gamma }_{32}={\gamma }_{3d}=\Gamma$, ${\Gamma }_{21}={\gamma }_{2d}=0.01\Gamma$, $\alpha l=107\Gamma$, ${\Delta }_{c}=0$ and $\Phi =0$. This figure shows that the proper choices of the intensity of the acoustic field and the static magnetic field prepare the system to transmit the probe field with different polarization plane rotation. It is seen that the polarization direction of the transmitted field can cover a wide range of the MOR angle from zero to 90 degrees for different values of ${\Delta }_{B}$ and ${\Omega }_{s}$.

**Figure 8:**
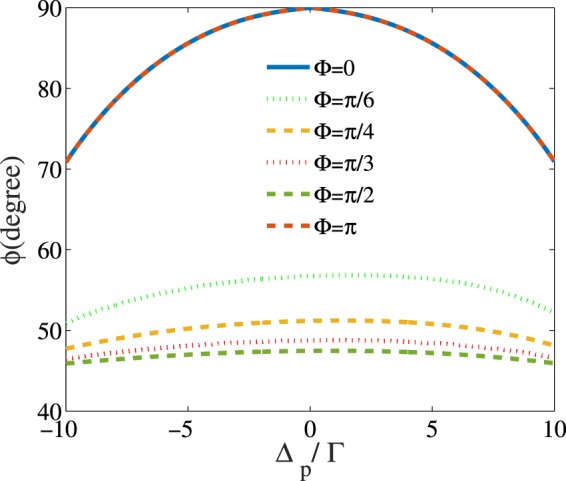
MOR angle of the polarization direction of the linearly polarized probe field versus detuning of the probe field for different values of the relative phase of the applied fields. The taken parameters are ${\Omega }_{p}=0.01\varGamma$, ${\Omega }_{s}=17\Gamma$, ${\Delta }_{B}=17\Gamma$, ${\Gamma }_{31}={\Gamma }_{32}={\gamma }_{3d}=\Gamma$, ${\Gamma }_{21}={\gamma }_{2d}=0.01\Gamma$, $\alpha l=107\Gamma$ and ${\Delta }_{c}=0$.

Another parameter for controlling the MOR angle is the relative phase of the applied fields, which can be simply changed by electro-optical phase modulators. It is well known that the optical properties of a closed-loop system, in multi-photon resonance condition, depend on the relative phase of the applied fields^42^. Thus, it is expected that the relative phase of the applied field becomes a useful tool to impose desired rotation on the polarization direction of the probe field. In Fig. 8, the MOR angle of the probe field is presented versus ${\Delta }_{p}$ for different values of the relative phase of the applied fields. The taken parameters are ${\Omega }_{p}=0.01\Gamma$, ${\Omega }_{s}=17\Gamma$, ${\Delta }_{B}=17\Gamma$, ${\Gamma }_{31}={\Gamma }_{32}={\gamma }_{3d}=\Gamma$, ${\Gamma }_{21}={\gamma }_{2d}=0.01\Gamma$, $\alpha l=107\Gamma$ and ${\Delta }_{c}=0$. It is seen that the polarization plane of the probe field takes different angles for different values of the relative phase so that at $\Phi =0$ and $\Phi =\pi$, it is rotated by 90 degrees at the probe field resonance.

To have a good insight into the effect of the parameters, we present the analytical solution for the transition coherences ${\rho }_{21}$ and ${\rho }_{31}$ in the weak probe field approximation (${\Omega }_{p\pm }\ll \varGamma$) derived from Eq. (9) as follows

10 $${\rho }_{21}=\frac{\mathrm{2(3}i\Gamma +2{\Delta }_{p}-2{\Delta }_{B})({\Delta }_{p}^{2}-{\Delta }_{B}^{2})}{A}{\Omega }_{p-}-\frac{\mathrm{2(3}i\Gamma +2{\Delta }_{p}-2{\Delta }_{B})|{\Omega }_{s}{|}^{2}}{A}{\Omega }_{p-}+\frac{-\mathrm{4(}{\Delta }_{p}^{2}-{\Delta }_{B}^{2}){\Omega }_{s}^{\ast }+\mathrm{4|}{\Omega }_{s}{|}^{2}{\Omega }_{s}^{\ast }}{A}{\varOmega }_{p+},$$

11 $${\rho }_{31}=\frac{\mathrm{2(}i\Gamma +2{\Delta }_{p}+2{\Delta }_{B})({\Delta }_{p}^{2}-{\Delta }_{B}^{2})}{A}{\Omega }_{p+}-\frac{\mathrm{2(}i\Gamma +2{\Delta }_{p}+2{\Delta }_{B})|{\Omega }_{s}{|}^{2}}{A}{\Omega }_{p+}+\frac{-\mathrm{4(}{\Delta }_{p}^{2}-{\Delta }_{B}^{2}){\Omega }_{s}+\mathrm{4|}{\Omega }_{s}{|}^{2}{\Omega }_{s}}{A}{\Omega }_{p-},$$

where $$A\mathrm{=(3}\Gamma -2i({\Delta }_{p}-{\Delta }_{B}))({\Delta }_{p}^{2}-{\Delta }_{B}^{2})(\Gamma -2i({\Delta }_{p}+{\Delta }_{B}))+(-3{\Gamma }^{2}+8{\Delta }_{p}(i+{\Delta }_{p})+4i{\Delta }_{B}-8{\Delta }_{B}^{2})|{\Omega }_{s}{|}^{2}-\mathrm{4|}{\Omega }_{s}{|}^{4}$$.

These equations show the explicitly analytical relation of ${S}_{+}$ and ${S}_{-}$ with the externally applied control parameters, so they let us control the MOR angle of the polarization direction of the probe field by properly adjusting the intensity of the acoustic field and the static magnetic field. The first terms in Eqs. (10) and (11) are the direct responses of the medium to the left- and right- circular components of the probe field via one-photon transition, respectively. The second terms show the cross-Kerr effect through three-photon transitions $\mathrm{|1}\rangle \,\mathop{\longrightarrow }\limits^{{\Omega }_{p-}}\,\mathrm{|2}\rangle \,\mathop{\longrightarrow }\limits^{{\Omega }_{s}}\,\mathrm{|3}\rangle \,\mathop{\longrightarrow }\limits^{{\Omega }_{s}^{\ast }}\,\mathrm{|2}\rangle$ and $\mathrm{|1}\rangle \,\mathop{\longrightarrow }\limits^{{\Omega }_{p+}}\,\mathrm{|3}\rangle \,\mathop{\longrightarrow }\limits^{{\Omega }_{s}^{\ast }}\,\mathrm{|2}\rangle \,\mathop{\longrightarrow }\limits^{{\Omega }_{s}}\,\mathrm{|3}\rangle$ for the left- and right- circular polarization of the probe field, respectively. The third ones correspond also to the cross-Kerr effect, but each of them is proportional to the field that excites the transition of the other side. This effect arises through a two-photon transition $\mathrm{|1}\rangle \,\mathop{\longrightarrow }\limits^{{\Omega }_{p+}}\,\mathrm{|3}\rangle \,\mathop{\longrightarrow }\limits^{{\Omega }_{s}^{\ast }}\,\mathrm{|2}\rangle$ and a four-photon transition $\mathrm{|1}\rangle \,\mathop{\longrightarrow }\limits^{{\Omega }_{p+}}\,\mathrm{|3}\rangle \,\mathop{\longrightarrow }\limits^{{\Omega }_{s}^{\ast }}\,\mathrm{|2}\rangle \,\mathop{\longrightarrow }\limits^{{\Omega }_{s}}\,\mathrm{|3}\rangle \,\mathop{\longrightarrow }\limits^{{\Omega }_{s}^{\ast }}\,\mathrm{|2}\rangle$ for the left circular component, while the cross-Kerr effect appears for the right- component via a two-photon transition $\mathrm{|1}\rangle \,\mathop{\longrightarrow }\limits^{{\Omega }_{p-}}\,\mathrm{|2}\rangle \,\mathop{\longrightarrow }\limits^{{\Omega }_{s}}\,\mathrm{|3}\rangle$ and a four-photon transition $\mathrm{|1}\rangle \,\mathop{\longrightarrow }\limits^{{\Omega }_{p-}}\,\mathrm{|2}\rangle \,\mathop{\longrightarrow }\limits^{{\Omega }_{s}}\,\mathrm{|3}\rangle \,\mathop{\longrightarrow }\limits^{{\Omega }_{s}^{\ast }}\,\mathrm{|2}\rangle \,\mathop{\longrightarrow }\limits^{{\Omega }_{s}}\,\mathrm{|3}\rangle$. The results from Eqs. (10) and (11) are in good agreement with the obtained numerical results.

**Figure 9:**
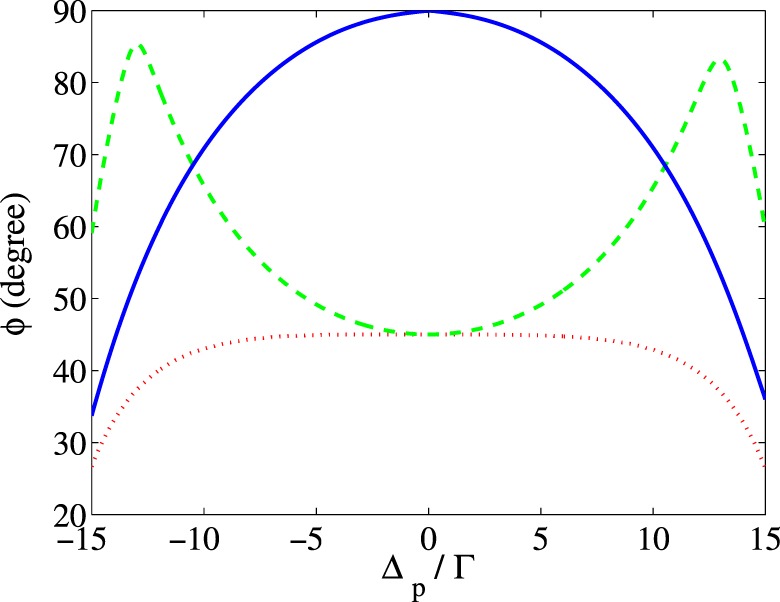
Contribution of the direct response of the medium (dotted) to the probe field, cross-Kerr effect (dashed) and their combination (solid) to the MOR angle versus detuning of the probe field. The taken parameters are those used in Fig. 2.

Now, we are interested in studying the effect of the above-mentioned optical phenomena on the MOR. The contribution of the direct response of the medium (dotted), the cross-Kerr effect (dashed) and their combination (solid) to the MOR angle of the polarization direction are depicted in Fig. 9 versus the detuning of the probe field. Figure 9 demonstrates the major role of the nonlinearity caused by the acoustic field through the cross-Kerr effect on enhancing the MOR angle, while the direct linear response of the medium can rotate the polarization direction of the probe field only 45 degrees.

**Figure 10:**
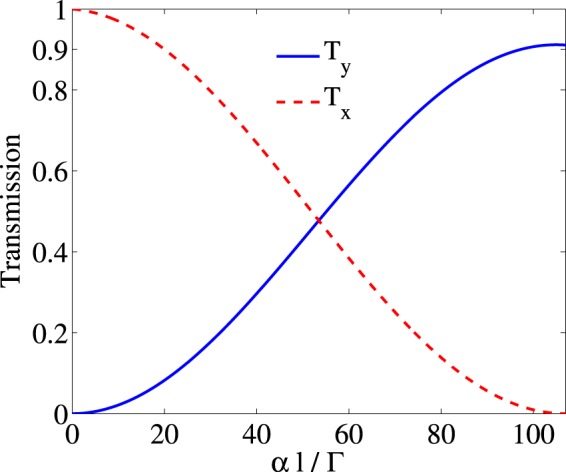
Intensity of the transmission in direction $\hat{x}$ (dashed) and ${T}_{y}$ (solid) versus $\alpha l$. The taken parameters are ${\Omega }_{p}=0.01\Gamma$, ${\Omega }_{s}=17\varGamma$, ${\Delta }_{B}=17\varGamma$, ${\Gamma }_{31}={\Gamma }_{32}={\gamma }_{3d}=\Gamma$, ${\Gamma }_{21}={\gamma }_{2d}=0.01\Gamma$, ${\Delta }_{c}=0$, ${\Delta }_{p}=0$ and $\Phi =0$.

The absorption coefficient, $\alpha l$, which is related to the length of the medium and also the density of atoms has a major role in switching the polarization direction of the output probe field from $\hat{x}$ direction to $\hat{y}$ direction. Figure 10 illustrates the intensity of transmission of the probe field in $\hat{x}$ (dashed) and $\hat{y}$ (solid) directions as a function of $\alpha l$ for the case that the probe field is at resonance ${\Delta }_{p}=0$. The other used parameters are ${\Omega }_{p}=0.01\Gamma$, ${\Omega }_{s}=17\Gamma$, ${\Delta }_{B}=17\Gamma$, ${\Gamma }_{31}={\Gamma }_{32}={\gamma }_{3d}=\Gamma$, ${\Gamma }_{21}={\gamma }_{2d}\mathrm{=0.01}\Gamma$, ${\Delta }_{c}=0$ and $\Phi =0$. It is seen that ${T}_{x}$ attenuates to a negligible value by increasing the $\alpha l$, while ${T}_{y}$ enhances and reaches its maximum value at $\alpha l=107\Gamma$. Since the two so-called electromagnetic modes, TE and TM, are perpendicular, our presented scheme can be used as a polarization converter for switching TM/TE modes.

## Conclusion

In summary, the nonlinear MOR of a linearly polarized microwave probe field was investigated after passing through many NV centers embedded in a high-Q single-crystal DMR. We established a three-level closed-loop system from the ground states of the NV center by the mechanism of the strain-mediated coupling in exciting the optically dipole forbidden transition in the presence of a static magnetic field. It was shown that by applying an acoustic field and a static magnetic field, difference between the refractive indices of the circular components of the probe field increases and the birefringence due to the cross-Kerr effect is induced in the system. We obtained a large intensity for the transmitted probe field and it was demonstrated that the acoustic field can enhance the MOR angle to 90 degrees through the cross-Kerr effect. Moreover, we showed that the MOR is sensitive to the relative phase of the applied fields. The physical mechanism was explained using the analytical expressions. Our scheme can be used as a polarization converter for efficient switching TE/TM modes in optical communications, polarization spectroscopy, the depolarization backscattering lidar and precision measurements.
